# Prospects and Challenges of Using Machine Learning for Academic Forecasting

**DOI:** 10.1155/2022/5624475

**Published:** 2022-06-17

**Authors:** Edeh Michael Onyema, Khalid K. Almuzaini, Fergus Uchenna Onu, Devvret Verma, Ugboaja Samuel Gregory, Monika Puttaramaiah, Rockson Kwasi Afriyie

**Affiliations:** ^1^Department of Mathematics and Computer Science, Coal City University, Enugu, Nigeria; ^2^National Center for Cybersecurity Technologies (C4C), King Abdulaziz City for Science and Technology (KACST), Riyadh 11442, Saudi Arabia; ^3^Department of Computer Science, Ebonyi State University, Abakiliki, Nigeria; ^4^Department of Biotechnology, Graphic Era Deemed to be University, Dehradun, Uttarakhand, India; ^5^Department of Computer Science, Michael Okpara University of Agriculture, Umuahia, Nigeria; ^6^Department of Machine Learning, BMS College of Engineering, Bengaluru, India; ^7^Department of Information and Communication Technology, Dr. Hilla Limann Technical University, WA, Ghana

## Abstract

The study examines the prospects and challenges of machine learning (ML) applications in academic forecasting. Predicting academic activities through machine learning algorithms presents an enhanced means to accurately forecast academic events, including the academic performances and the learning style of students. The use of machine learning algorithms such as K-nearest neighbor (KNN), random forest, bagging, artificial neural network (ANN), and Bayesian neural network (BNN) has potentials that are currently being applied in the education sector to predict future events. Many gaps in the traditional forecasting techniques have greatly been bridged by the use of artificial intelligence-based machine learning algorithms thereby aiding timely decision-making by education stakeholders. ML algorithms are deployed by educational institutions to predict students' learning behaviours and academic achievements, thereby giving them the opportunity to detect at-risk students early and then develop strategies to help them overcome their weaknesses. However, despite the benefits associated with the ML approach, there exist some limitations that could affect its correctness or deployment in forecasting academic events, e.g., proneness to errors, data acquisition, and time-consuming issues. Nonetheless, we suggest that machine learning remains one of the promising forecasting technologies with the power to enhance effective academic forecasting that would assist the education industry in planning and making better decisions to enrich the quality of education.

## 1. Introduction

Machine learning is rapidly gaining influence in the education industry and can possibly strengthen many important areas of teaching and learning, research, and decision-making [1]. It is about mimicking humans in terms of both reasoning and behaviour [2, 3]. Naïve Bayes, decision tree, and many other related algorithms have prognostic capabilities that are of great interest to medical educators and [4] and are being used to facilitate learning [5]. Machine learning models learn from experiences or observations and then become very active to achieve human-like actions [6, 7]. Investigations have shown that ML has forecasting capabilities by gaining an understanding of each student's strengths and weaknesses, as well as reasons for why they may be struggling in the classroom [8, 9].

Forecasting of learning outcomes using the machine learning approach can help educational institutions to understand the learning patterns and behaviours of their students, which can be useful in improving school policies, curriculum preparations, and teacher methodology [10]. Indeed, machine learning algorithms offer opportunities for organizations, including educational institutions to achieve minimal forecast errors (accuracy) and improve their decision-making process and operational performances [11]. ML provides improvements that help reduce forecast mistakes or errors that have been a source of concerns to many forecasters and their clients in recent times. The inaccuracy in forecasting often causes major problems to systems and could lead to poor decision-making and system failure. With machine learning models that monitor learners' progress and recommend next steps have been achieved, and learning analytics are also made possible to accommodate special learners and those that require more attention or personalized teaching. This study reviews the benefits of ML and its shortcomings when deployed for academic forecasting. The study would go a long way to assist forecasters and education authorities in understanding the emerging prospects and challenges of machine learning and how best to apply it in the education sector to minimize student's failures and to enhance the decision-making process. This investigation adds to the body of information about the educational applications of machine learning and artificial intelligence.

## 2. Related Literature

Several researchers [7, 10, 12–19] have highlighted the growing application and usefulness of machine learning as a tool for forecasting of events in the academic sector. Similarly, a study by Elhaj et al. [13] proved the exactness of the KNN algorithm in detecting the student's learning style, which is fast becoming helpful in training at-risk students.

Halde [20] gave analysis of various ways in which machine learning can be applied by educational institutions to predict students' performance and the critical characteristics that have to be considered while making such predictions. The researcher discovered that ML enhances more precise and accurate prediction of academic performances, but features such as learning styles, motivation, and many other factors relating to students' learning have to be considered to achieve accuracy. Essinger and Rosen [21] presented a system to aid students' motivation and interests in problem solving using machine learning.

Dhanabal and Chandramathi [22] and Itoh et al. [23] developed a model based on the machine learning algorithm with the capacity to accurately forecast students' academic outputs based on their historical or previous record of performances. They achieved success in the study as students' academic standings were predicted, thereby enabling those at risk to receive some help that enhanced their performances. Research on ML is fast growing, but there are gaps in the literature regarding its prospects and challenges in academic forecasting in recent times as most existing studies seemed to have been conducted years ago. Thus, this study provides updated knowledge in this regard. The summary of the review is illustrated in Table 1.

## 3. Overview of Machine Learning

Machine learning is about training machines to learn certain behaviours or traits and then think and make decisions based on the learned behaviour. It remains one of the promising areas of research to solve many societal problems and can be classified as supervised and unsupervised learning techniques. Through the use of an unsupervised method, you may see if a system can derive data and inferences even when there are not any results or training data. While in the supervised learning field, for each observation of the predictor *x*_*i*_, *i* = 1,…, *n* there is a related outcome *y*_*i*_ [7]. Supervised algorithms facilitate the understanding of the learning history of the system and the correctness of the output for future analysis. Machine learning can be applied in different areas, including DNA sequence classification, image processing such as face detection, speech recognition aka natural language processing, security, search engine algorithms, and academic forecasting [34, 35].

According to trueinteraction.com [36] and Edeh et al. [37], predictions based on machine learning can be reliable if the dataset is properly trained and the validation of the model is right. Indeed, the prospects of ML are huge, and as the education industry embraces new realities, ML together with other emerging technologies could play a bigger role in learning analytics, e-learning, students' examinations and performance prediction and tracking of teaching, and learning progress. Several machine learning algorithms such as multilayer perceptron (MLP) and CART regression trees (CART) have different potentials which are of great interest to stakeholders in the academic sector. For machine learning to be able to understand a dataset, it has to be trained and also pass through different processes to fully comprehend such data and be able to interpret and enhance prediction or any other expected decisions. This study looks at the pros and cons associated with machine learning use in academic forecasting compared to other forecasting methods. Consequently, Table 2 highlights the comparison between the machine learning-based forecasting and the traditional forecasting technique.

**Figure 1:**
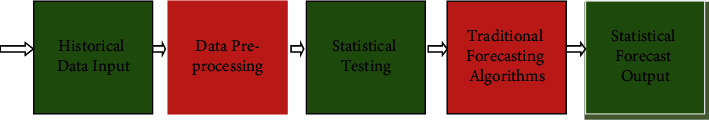
The traditional forecasting approach [41].

**Figure 2:**
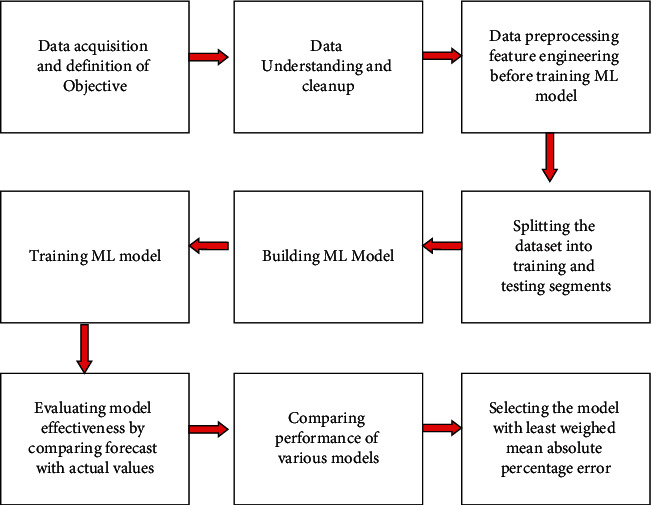
Machine learning forecasting approach [41].

The comparison between traditional and machine learning forecasting techniques is further explained in Figures 1 and 2 adopted from the Genpact.com website.

Figure 1 shows the stages applied in the traditional forecasting technique. While Figure 2 depicts different stages that forecasting through machine learning goes through before generating an output. As it can be seen, Figure 2 contains more robust or rigorous procedures that actually improve the accuracy of the output that is produced compared to that of the traditional method shown in Figure 1.

## 4. Prospects of Forecasting Academic Events with Machine Learning

The evolution of artificial intelligence has increased the use of machine learning as a means to achieve academic prediction precision and timeliness [42]. Many educational institutions are utilising machine learning algorithms and their accompanying features to predict possible future events and outcomes to aid decision-makers in making the best possible decisions. ML has become a good alternative to traditional statistical methods of forecasting [39]. Some of the benefits of forecasting with ML are explained in the following section.

### 4.1. Pattern Recognition

Machine learning models can be trained with large volumes of data which it tends to understand later and figure out different trends and patterns in the data that ordinarily would prove very difficult for humans to comprehend. Decision-makers can examine trends and seasonal realities that affect their organization's performance by understanding and analyzing these patterns [43]. In academics, learners' pattern is closely monitored thereby enabling educators to teach better and also diagnose learning difficulties of students with a view to assist vulnerable students.

### 4.2. Reduced Human Intervention

Since machine learning has the ability to learn and interpret the learnt dataset, forecasters often do not need to interfere much with the prediction process. This is because the machine learning algorithms can automatically make predictions using the pattern of data in the set. This act minimizes human bias or interferences experienced in the traditional forecasting approach [44]. As it can be seen in the functioning of antivirus software, they do not require human intervention to recognize potential attacks or threats in the computer before detecting or blocking them. It does that autonomously and automatically.

### 4.3. Accurate Academic Forecasting

Machine learning algorithms gain, learn, and acquire experiences overtime, which help them become better and better in their prediction ability. The use of the ML forecasting method can improve the accuracy and efficiency of academic predictions compared to other prediction techniques such as time series [39]. It makes it easier to generate data to identify at-risk students and then develop diagnostic measures to help them overcome their weaknesses [45]. Forecasting accuracy is vital as it helps educational institutions to make adequate planning and preparations towards future events which include risks, thereby giving them enough time to find measures to mitigate its effects. However, machine learning algorithms depend on several elements, including the quality and speed with which the machine learns and improves its performance, which might affect its precision. Predictive outcomes may be affected by changes in a model variable [46].

### 4.4. Forecasting of Large Volume of Education Data

In this digital era with high demand for a wide range of education data, machine learning is the right method to analyze or interpret and deal with a broad range of data and can also respond to changes in data and the environment. Large datasets can easily be mined and meaningful patterns predicted with minimum use of resources [47].

### 4.5. Continuous Improvement

The ability of machine learning to continuously learn and adapt to changing situations presents advantages in the academic sector. This is because the education sector is dynamic, and changes are always expected most times. The ML algorithm is able to adjust quickly and accommodate these emerging changes and improve itself rapidly [48]. Whenever it comes to producing predictions, machine learning is a better option because of its speed.

### 4.6. Academic Cost Reduction

Machine learning generally relies on very strong assumptions about the statistical stability of the environment. The prediction of expected future happenings would enable educational institutions to plan their admissions, policies, and priorities thereby enhancing their preparedness and reducing cost [49]. Accurate predictions via machine learning go a long way to assist policy makers and other education stakeholders to realign their focus in line with trends and also save cost that could have emanated from poor decision-making as a result of lack of accurate prediction.

### 4.7. Management of Unexpected Closure of Schools

Machine learning can be relied on by education institutions to help them interpret data relating to emergencies especially as it concerns crisis that can lead to shutdown of schools. During the recent COVID-19 crisis, machine learning played an important role in interpreting data that were relevant in reopening of schools and development of strategies for post-COVID-activities.

**Figure 3:**
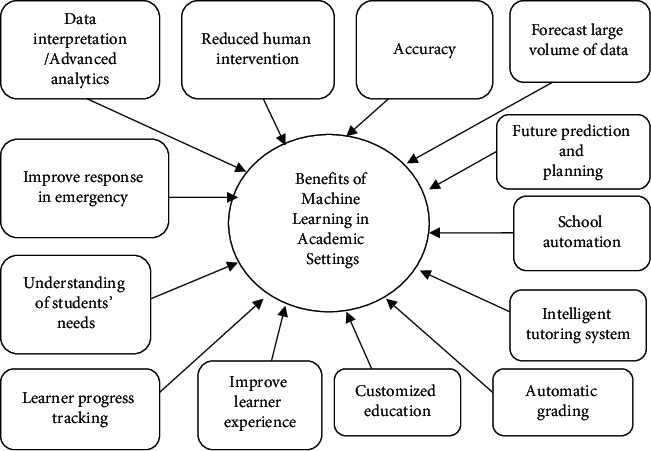
Benefits of machine learning in academic setting and forecasting.

The various prospects and benefits of machine learning in academic forecasting and other areas in education are summarized in Figure 3.

## 5. Challenges of Forecasting with Machine Learning

There are flaws in machine learning. Similar to other machine and human forecasting methods, machine learning has its own set of flaws. However, it might be claimed that machine learning presents significantly fewer obstacles than traditional forecasting methods [48]. For example, machine learning does not totally depend on historical data to make predictions, it learns from experiences and understands patterns, which may not have existed earlier but are learnt during the training of the model. However, machine learning has many limitations in performance evaluation [49]. Some of the challenges associated with the use of ML for forecasting are categorized as follows:

### 5.1. Proneness to Errors

Evidence has shown that ML is autonomous but highly susceptible to both machine and human errors [13]. For instance, if the right amount of data is not trained in the dataset, biased predictions can be made and generalized [50]. This is because the quality of prediction would largely depend on the correctness of the training dataset. Prediction errors in ML often appear which are very difficult to diagnose and correct because they require rigorous underlying complexities of the algorithms and associated processes [43].

### 5.2. Data Acquisition

ML algorithms rely on data to learn and predict academic events. It is data hungry. However, the acquisition of these data is not easy, but the larger the data, the better the machine learning and prediction reliability [43].

### 5.3. Time Factors

Machine learning algorithms require sufficient time to train and learn to be able to function effectively. This could lead to time wastage. The ML forecasting technique, on the other hand, necessitates patience and time to assure accuracy. The time complexity could increase depending on the size of data. The more the data are, the more time would be required.

### 5.4. Verification Challenges

Sometimes, it is difficult to verify certain facts that are not included in the historical data, which means that ML predictions may not be accurate in certain cases. It could also take some time for the model to be trained or master the dataset for effective decision.

## 6. Conclusion

Machine learning presents solutions to many limitations in the education sector, including academic forecasting. Not only does it offer intelligent and accurate academic predictions, but it also assists the teachers and educational institutions to understand their students' betterment and help them succeed. Though, many educational institutions in developing countries seem not ready to adopt machine learning approaches, but the changing realities in global education would leave them with no choice than to begin to strategize on how to adopt ML and other emerging technologies. Prediction through machine learning and other artificial intelligence solutions has the potential to change the narratives in the education sector, particularly the diagnosing of students' learning patterns and performance. Our future projects will focus on development of the ANN model for academic outcome prediction in secondary schools in Enugu Nigeria.

## Figures and Tables

**Table 1:** Summary of related works.

Authors	Outcome
Musso et al. [10]	The study successfully forecasted students' academic success one year ahead using the ANN based on cognitive and demographic traits
Hudson and Cristiano [7]	The results suggest that ML can generate dependable results in prediction
Elhaj et al. [13]	The study was empirical, and it showed the ability of KNN in prediction of learning patterns of students
Ahajjam et al. [24]	The paper provided AI-based solutions to track students' performance and was able to recommend diagnosis for the Moroccan students
Pranav et al. [25]	The paper provided evidence on the significance of AI in management of education data and decision-making
Lidia et al. [26]	The paper concluded that ML will be required more in the future because of the need to assist students to overcome learning difficulties and also enhance their productivity in learning
Phauk and Takeo [27]	The study recommended the use of the hybrid machine learning algorithm approach to solve misclassification issues and improve academic prediction accuracy
Onan and Korukoğlu [28]	The research proposed an ensemble method to feature selection that combines the results of numerous independent feature lists generated by various features that may be used in education
Onan [29]	The study provided a better approach for managing students' information system via ML
Hassen et al. [30]	The study showed that the student's success with the aid of machine learning can be monitored using their previous performance data before they engaged in the current program
Ibtehal [31]	The study affirmed the applicability of ML in education technology development and deployment
Feders and Anders [32]	They developed a smart algorithm that assessed the teaching methods of teachers and how it affects the understanding of their lessons by students in the class taking into consideration the former knowledge of students
Popenici and Kerr [33]	They examined the various implications of ML and other relevant AI-driven systems in higher education

**Table 2:** Machine learning-based forecasting vs. traditional forecasting technique.

Machine learning forecasting	Traditional forecasting
It gives more accurate predictions with minimal loss function [10, 13]	Forecast errors are more likely to occur [38]
The approach is more scientifically driven [26]	Suffers a lot from assumptions leading to subjective conclusions at times [7]
Very demanding in computation [39]	Less demanding computation
It is more prone to underfitting and overfitting issues [40]	Less prone to underfitting and overfitting issues
Focuses more on result or outcome, but silent relationships among variables.	Relationship between variables are often highlighted
Highly recommended in applications where the goal is to learn from datasets with a large number of characteristics [41]	Suitable in univariate applications often meant to assess and summarize data.
It can work with massive data	It works with limited or historical data

## Data Availability

All data have been cited within the paper.
